# Molecular epidemiological and spatiotemporal analysis of lumpy skin disease outbreaks in cattle from Karnataka, India

**DOI:** 10.3389/fcimb.2025.1596973

**Published:** 2025-06-24

**Authors:** Manjunatha Reddy Gundallahali Bayyappa, Chandana Ramesh Uma, Shraddha Bijalwan, Sunil Tadakod, Sudeep Nagaraj, Megha Naragund, Sai Mounica Pabbineedi, Chethan Kumar Harlipura Basavarajappa, Baldev Raj Gulati

**Affiliations:** Capripoxvirus Lab, Veterinary Pathology, Indian Agricultural Research Institute (ICAR)-National Institute of Veterinary Epidemiology and Disease Informatics (NIVEDI), Bengaluru, Karnataka, India

**Keywords:** cattle, diagnosis, epidemiology, lumpy skin disease, risk factors, spatial clustering, vaccination

## Abstract

**Introduction:**

Lumpy Skin Disease (LSD), is a re-emerging transboundary disease of cattle, which has severely impacted India’s livestock population. This study investigates its molecular epidemiology, spatial patterns, and risk factors in Karnataka from 2021–2024.

**Methodology:**

In this study, total of 1,353 different types of samples from 451 suspected LSD cases were analyzed using PCR. Spatial clustering was assessed using QGIS and GeoDa. Risk factor analysis and phylogenetic studies were performed to identify susceptible groups and viral lineages.

**Results:**

Among the analyzed samples, scabs showed the highest positivity rate at 83.6%, followed by nasal swabs at 80.9%, and blood samples at 76.9%, underscoring the utility of nasal swabs as a simple and effective non-invasive clinical sample for diagnosis and clinical surveillance. Spatial analysis conducted with QGIS (v3.32.2) and GeoDa (v1.22) identified strong spatial autocorrelation and consistent clustering of cases in densely populated livestock areas, driven by environmental factors and mechanical vectors, including blood-feeding arthropods like flies (*Stomoxys calcitrans*, *Haematobia irritans*) and ticks (*Rhipicephalus* and *Amblyomma* species). Vaccination campaigns achieved near 100% coverage in most districts by 2022, substantially reducing LSD-related mortality rate from 1.45% to negligible levels in 2024. Risk factor analysis identified cattle aged 1 to 5 years and females as most susceptible, with exotic breeds exhibiting 2.1–2.6 times higher odds of infection than indigenous breeds. Molecular studies unveiled a genetically stable lumpy skin disease virus strain unique to Karnataka, consistent with the low mutation rate of DNA viruses.

**Discussion:**

These findings emphasize that, despite vaccination success, persistent hotspots and vulnerable cattle groups remain. Therefore, region-specific strategies such as targeted vector control, robust molecular surveillance, and GIS-based early outbreak alerts are necessary for LSD control in endemic regions. This study also highlights how the integration of combining molecular diagnostics, epidemiological modeling, and spatial analytics can strengthen LSD surveillance and redefine disease control strategies, ultimately mitigating its socio-economic impact.

## 1 Introduction

Lumpy skin disease (LSD) is a re-emerging, economically significant, non-zoonotic, vector-borne viral disease affecting bovines. It is caused by the lumpy skin disease virus (LSDV), a member of the *Poxviridae* family and *Chordopoxvirinae* subfamily, primarily infecting *Bos taurus* (cattle) and *Bubalus bubalis* (buffalo) (Sudhakar et al., 2025). The disease manifests clinically with nodular skin lesions, typically 0.5 to 3 cm in diameter, accompanied by systemic symptoms such as anorexia, depression, reproductive disorders, infertility in both sexes, swollen lymph nodes, and reduced milk production due to stress (Abutarbush Sameeh, 2017). Transmission predominantly occurs via mechanical vectors, which facilitate the external transfer of pathogens without serving as biological hosts. Identified vectors include *Stomoxys calcitrans* (stable fly), *Aedes aegypti* mosquitoes (Chihota et al., 2001; Haegeman et al., 2023), and tick species belonging to the genera *Rhipicephalus* and *Amblyomma* (Tuppurainen et al., 2013; Lubinga et al., 2015). No evidence currently supports biological transmission involving virus replication within the vector. Although direct contact and fomite transmission are considered secondary routes, they may still contribute to disease spread (Sprygin et al., 2019).

Accurate and timely diagnosis is crucial for effective disease management. While clinical symptoms provide preliminary indications, laboratory confirmation is essential. The choice of diagnostic sample plays a significant role in detection accuracy. Immunological assays typically utilize serum samples, whereas molecular techniques rely on nasal swabs, blood, or scabs for LSDV detection. Conventional polymerase chain reaction (PCR), real-time PCR, and restriction fragment length polymorphism (RFLP) analysis are widely employed for precise diagnosis (Sameea Yousefi et al., 2017). Among these, conventional PCR is a widely used and cost-effective method. Commonly used primer sets target the *P32* envelope protein gene (*LSDV074*, 192 bp) and the fusion protein gene (*LSDV117*, 472 bp), along with *ORF036*, which encodes the RNA polymerase 30 kDa protein (*RPO30*), enhancing both detection sensitivity and specificity by targeting multiple genomic regions (Shchelkunov et al., 2005; Sreedevi and Rajesh, 2021).

The geographic expansion of LSD further underscores its evolving epidemiology. First identified in Zambia in 1929, the disease became endemic in parts of Africa before spreading to the Middle East, Europe, and Asia, with rapid expansion occurring post-2013. In India, LSD was first reported in August 2019 in Odisha, particularly in Mayurbhanj, Bhadrak, and Kendrapara districts (Sudhakar et al., 2020). Given its high transmissibility and economic impact, the World Organisation for Animal Health (WOAH) has designated LSD as a transboundary and notifiable disease, warranting stringent control measures (Reddy et al., 2024).

The global significance of LSD is amplified by its potential economic repercussions. Asia accounts for approximately 40% of the global cattle and buffalo population, with India alone housing 300 million bovines, followed by China (90 million) and Pakistan (85 million) (Roche et al., 2021). As a leading global beef exporter, India shipped approximately 527 tons in 2018. The country also possesses the world's largest livestock population, with 536.76 million animals, representing 15% of the global total. According to the 2021 Basic Animal Husbandry (BAH) Statistics, the livestock sector contributes 6.20% to India's gross domestic product (GDP) and 31.0% to the agricultural GDP, underscoring its economic importance (Naidu et al., 2025).

The recent outbreaks of LSD in India have highlighted the urgency for effective control measures. The 2019 outbreak in Odisha reported morbidity rates ranging from 0.75% to 14.04%, with Bhadrak district experiencing the highest incidence and Kendrapara district the lowest. The outbreak coincided with the monsoon season, with cases emerging in August and persisting thereafter (Roche et al., 2021). As of December 2022, LSD had been reported in 30 districts of Karnataka (Prabhu et al., 2023). By 2022, India had recorded approximately three million LSD infections in cattle, resulting in a mortality rate of nearly 6% and 155,000 fatalities (Kumar and Tripathi, 2022). To combat the outbreak, a nationwide vaccination campaign was initiated, successfully immunizing 181.93 million cattle by December 2023. Karnataka ranked third in vaccine coverage, implementing a large-scale vaccination program using a heterologous Goat pox vaccine with an efficacy of 70%–80%, significantly reducing mortality. The vaccination initiative, costing Rs 2,729 million, effectively prevented estimated economic losses of Rs 58,930 million, demonstrating its cost-effectiveness and crucial role in controlling LSD (Molla et al., 2017).

The ongoing spread of LSD and its economic consequences necessitate continued surveillance, research, and vaccination efforts to mitigate its impact. This study aims to analyze epidemiological trends, assess risk factors, and evaluate spatial-temporal distribution using geographic information systems (GIS) to map high-risk zones in Karnataka. Additionally, molecular diagnostics were employed to determine the most effective sample type (nasal swabs, blood, or scab samples) for LSDV detection, thereby enhancing early diagnosis and disease monitoring. These findings generate actionable insights to inform practical disease control strategies and region-specific policy measures for managing LSD in endemic and high-risk regions effectively.

## 2 Methodology

### 2.1 Epidemiology and disease impact

The state of Karnataka comprises 31 districts, which according to the Karnataka State Remote Sensing Applications Centre are classified into ten distinct agro-climatic zones namely, North-Eastern Transition Zone, North-Eastern Dry Zone, Northern Dry Zone, Central Dry Zone, South-Eastern Dry Zone, Southern Dry Zone, Southern Transition Zone, Northern Transition Zone, Hilly Zone, and Coastal Zone. The sampling strategy was designed to ensure broad representation across these agro-climatic zones, facilitating a comprehensive epidemiological analysis of the Lumpy Skin Disease Virus (LSDV) throughout the state (**Supplementary Table 1**).

Geographical data were used to investigate the epidemiology of Lumpy Skin Disease (LSD), assess the impact and severity of outbreaks, and evaluate the effectiveness of vaccination programs in controlling the disease in Karnataka from 2021 to 2024. Retrospective epidemiological data, including district-wise LSD cases, outbreaks, deaths, and vaccination details, were obtained from the Department of Animal Husbandry and Veterinary Services in Karnataka. Spatial analysis was conducted using Quantum Geographic Information System (QGIS) software (version 3.32.2) to create district-level maps illustrating the cattle population, mortality rate, and vaccination coverage. The mortality rate was measured to assess the impact of Lumpy Skin Disease on the cattle population.

The mortality rate and vaccination coverage were calculated using the following formulas:


$$Mortality\;rate\;\left(\%\right)=\;\frac{Total\;number\;of\;LSD\;related\;deaths}{Total\;cattle\;population\;at\;risk}\times 100$$

$$Vaccination\;coverage\;\left(\%\right)=\;\frac{Total\;number\;of\;vaccinated\;cattle}{Total\;susceptible\;cattle\;population}\times 100$$

The Case Fatality Rate (CFR) was analyzed to assess the severity of Lumpy Skin Disease (LSD) across different agro-climatic zones for the periods 2021–2022, 2022–2023, and 2023–2024. CFR was determined by calculating the proportion of confirmed cases that resulted in mortality, providing a quantitative measure of disease severity. The CFR was computed using the following formula:


$$Case\;fatality\;rate\;\left(CFR\;\%\right)=\;\frac{Total\;number\;of\;LSD\;deaths}{Total\;confirmed\;LSD\;cases}\times 100$$

### 2.2 Exploratory spatial data analysis

Exploratory Spatial Data Analysis (ESDA) was employed to assess the spatiotemporal distribution and patterns of LSD cases in Karnataka from 2021 to 2024. The temporal progression of the disease was analyzed through spatial cluster analysis using GeoDa software version 1.22. Thematic maps were generated to illustrate the district-wise distribution of LSD cases over three time periods: 2021-2022, 2022-2023, and 2023-2024, utilizing data from the Department of Animal Husbandry and Veterinary Sciences, Karnataka.

Temporal analysis was performed by monitoring spatial clustering trends over successive years (2021–2022, 2022–2023, and 2023–2024), enabling an understanding of disease progression across districts. A spatial weight matrix was used to assess the relationships between the 31 districts, employing a queen contiguity matrix to define neighborhood interactions.

Moran's I statistic was used to assess the strength of spatial clustering and disease heterogeneity in the study area. The Moran's I value ranges from -1 to +1, where values near zero indicate random distribution, +1 signifies strong positive clustering, and -1 indicates strong negative clustering. Local Indicators of Spatial Association (LISA) clustering was performed to further identify spatial clusters and outliers. LISA categorizes districts into high-high, low-low, low-high, and high-low clusters, indicating varying patterns of spatial association. For further spatial evaluation, the Getis-Ord-Gi* statistic was used to measure local spatial autocorrelation and identify hot and cold spots of LSD cases.

### 2.3 Sample collection and ethical approval

During the LSD outbreak, field investigations were carried out with approval from the Department of Animal Husbandry and Veterinary Sciences, Karnataka. Clinical samples were collected from affected cattle in accordance with the guidelines set by the World Organisation for Animal Health (WOAH) from 21 districts across eight agro-climatic regions of Karnataka. Nasal swabs were collected by carefully inserting a sterile, flexible, fine-shafted swab into the nasopharyngeal cavity and gently rotating it to absorb secretions. The swabs were subsequently placed into sterile collection tubes containing 1 mL of viral transport medium (VTM). Additionally, 2–3 mL of blood was drawn via jugular vein puncture and collected in ethylene diamine tetra acetic acid (EDTA) vacutainers. Skin nodules or scabs from cattle exhibiting clinical signs of the disease were aseptically collected by first washing and cleaning the affected area with sterile saline and removing the hair using sterile scissors. The site was then punctured, and a portion of the nodule or scab was excised after disinfection with 70 percent isopropyl alcohol.

Additionally, a structured questionnaire was employed to gather essential information regarding the sampled animals, including age, sex, breed, and demographic details such as district and region. A total of 1,353 samples were collected from 21 districts across eight agro-climatic regions of Karnataka. To ensure sample integrity, all specimens were transported in insulated shipping containers with dry-ice packs, maintaining a strict cold chain. Samples were analyzed within 4–8 hours of receipt at the BSL-2+ virology laboratory, Indian Council of Agricultural Research-National Institute of Veterinary Epidemiology and Disease Informatics (ICAR-NIVEDI), under the approval of the Institute Animal Ethics Committee (No. NIVEDI/IAEC/2022/06), Bengaluru. All samples were subsequently stored at -20°C except for blood samples, which were preserved at 4°C.

### 2.4 Sample processing and genomic DNA extraction

The collected samples were thawed to room temperature before processing. Nasal swabs were vortexed thoroughly and aliquoted for extraction, while 150 μL of blood was suspended in 50 μL of 1X phosphate-buffered saline (PBS, pH 7.2). Approximately 30 mg of skin nodules or scab samples were finely chopped using a sterile Bard Parker blade, and a tissue homogenate was prepared by triturating the sample with 1 mL of chilled 1X PBS using a mortar and pestle. The total genomic DNA was extracted from the processed clinical samples using the DNeasy Blood and Tissue Kit (QIAGEN, Hilden, Germany), following the manufacturer's protocol. DNA concentration and purity were assessed using a Nanodrop spectrophotometer (NABI, Microdigital Co., Ltd., South Korea). The extracted DNA was stored at -20°C until further use. All biological materials were disposed of in accordance with the standard operating procedures (SOPs) established by the Institute Biosafety Committee (IBSC).

### 2.5 Molecular detection of LSDV

Diagnostic screening for the presence of lumpy skin disease virus (LSDV) was conducted on 1,353 clinical samples, including nasal swabs(n=451), whole blood (n=451), and skin nodules or scabs (n=451) from the same cattle. DNA was extracted and initially amplified for the Capripoxvirus-specific partial *P32* gene, followed by amplification of the LSDV-specific extracellular enveloped virion (EEV) glycoprotein gene (LSDV126) using respective forward and reverse primers, as detailed in **Table 1**.

**Table 1:** Shows the primers used for conventional PCR assays targeting various LSDV genes, including gene name, primer sequences, and expected amplicon size for confirmation of LSD.

Gene	Primer	Sequence (5′-3′)	Amplicon Size	Reference
Partial *P32*	*SGPP 32*-F	ACACAGGGGGATATGATTTTACC	237 bp	(Reddy et al., 2015)
	*SGPP 32*-R	ATACCGTTTTTCATTTCGTTAGC		
*LSD126*	LSDV-F	TAGAAAATGGATGTACCACAAATACAG	122 bp	(Pestova et al., 2018)
	LSDV-R	TTG TTA CAA CTC AAA TCG TTAG GTG		
Full-length *P32*	*B7*-F	AACACTCTCATTGGTGTTCGG	1012 bp	(Haegeman et al., 2020)
	*A95*-R	CACATGGCAGATATCCCATTA		
*GPCR* *(G-protein coupled receptor)*	*GPCR*-F	TTTATCAGCACTAGGTCATTATCT	1200 bp	(Zhou et al., 2012)
	*GPCR*-R	TATCACTCCCTTCCATTTTTAT		
*RPO30*	*RPO30*-F	ATAACCTACATGCATAAACAGAAG	840 bp	(Tulman et al., 2001)
	*RPO30*-R	ATACGAATCTACTTCATCACAAGA		

Conventional polymerase chain reaction (PCR) assay was performed using a Bio-Rad thermal cycler (Hercules, CA, USA) with a total reaction volume of 25 μL. The reaction mixture comprised 12.5 μL of PCR master mix (DreamTaq Green PCR Master Mix 2X, Thermo Scientific, Waltham, MA, USA), 1 μL each of forward and reverse primers (10 pmol/μL), 8.5 μL of nuclease-free water, and 50 ng of template DNA. Following amplification, PCR products were analyzed via agarose gel electrophoresis on a 1.5% agarose gel prepared in 1X Tris-acetate-EDTA (TAE) buffer, stained with ethidium bromide, and visualized under a UV transilluminator using a Gel Documentation System (Vilber Bio-Print, Vilber Lourmat, France).

### 2.6 Virus isolation

The Madin-Darby Bovine Kidney (MDBK) cell line was used for virus adaptation and isolation. Nasal swab samples, skin nodules, and scab tissue suspensions that tested positive by PCR were syringe-filtered using a 0.45 μm filter and subsequently used to infect a confluent monolayer of MDBK cells. Virus-infected MDBK cell lines were maintained in Minimum Essential Medium (MEM; HiMedia, Mumbai, India) supplemented with an antibiotic-antimycotic solution (1X; HiMedia, Mumbai, India) and 2% fetal bovine serum (FBS; MP Biomedicals, Santa Ana, CA, USA). The infected cells were incubated at 37°C in a 5% CO_2_ incubator (Thermo Scientific, Waltham, MA, USA) and monitored daily for five days to observe cytopathic effects (CPE). Following infection, the virus was harvested by subjecting it to three freeze-thaw cycles and stored at -80°C. The viral suspension was collected and passaged. Upon the appearance of CPE, genomic DNA was extracted from the harvested virus and confirmed by PCR.

### 2.7 Sequencing and phylogenetic analysis

PCR-positive amplicons of full-length genes (*P32*, *RPO30*, and *GPCR*), resolved by agarose gel electrophoresis, were excised and purified using the NucleoSpin Gel and PCR Clean-up Kit (Macherey-Nagel, Düren, Germany), following the manufacturer's instructions. The purified DNA was stored at -20°C and subsequently subjected to Sanger sequencing at Eurofins Genomics India Private Limited, Bengaluru, India. Sequencing data were analyzed and edited using GeneTool software (Informer Technologies, Inc.), and the sequences were submitted to GenBank database and are available under the accession numbers as follows: (*P32* gene: OR472969-OR472971, OR472973, OR472975, OR863389, OR602866, OR472977, OR699297, OR699298; *RPO30* gene: OP903442, OP903444-OP903458, OM362830, OM362845, OR863389, OR602866; *GPCR* gene: PP530467, PP530477, PP530466, PP530475, OR602866, OR863389, OR699291).

Comparative sequence analysis was performed using Capripoxvirus-associated nucleotide sequences of *P32*, *GPCR* (G-protein coupled receptor), and *RPO30* genes retrieved from GenBank, incorporating in-house Karnataka isolates, sequences from neighboring states, and other regions of India, as well as global LSDV strains, vaccine strains, and representative Sheep pox and Goat pox virus sequences. Multiple sequence alignment was performed using the MUSCLE algorithm in Molecular Evolutionary Genetics Analysis (MEGA) software, version 11. Phylogenetic relationships were inferred using the Maximum Likelihood method, employing the Tamura 3-parameter model for *P32* and *GPCR* genes and the Hasegawa-Kishino-Yano model for *RPO30*, with bootstrap analysis of 1000 replicates to assess branch reliability. The tree with the highest log-likelihood value was selected, and the percentage of replicate trees supporting each cluster was displayed alongside the branches. Initial heuristic search trees were automatically generated using the Neighbor-Joining (NJ) and BioNJ algorithms, based on pairwise distance matrices computed from the selected model, and the topology with the highest log-likelihood was retained. The constructed phylogenetic tree was visualized using Tree Explorer and further annotated and refined using Interactive Tree of Life (iTOL) v7.

### 2.8 Study design and statistical analysis

The collected samples were categorized based on the information collected during the outbreak on various epidemiological criteria. For the statistical analysis, age was classified into three groups: less than 1 year (n = 336), 1 year - 5 years (n = 498), and ≥ 5 years (n = 519). Sex-based stratification divided the samples into male (n = 528) and female (n = 825). Breed classification included local breeds (n = 576), exotic breeds (n = 570), and non-descript breeds (n = 207). Spatial classification based on agro-climatic zones included: North-Eastern Dry Zone (n = 147), Northern Dry Zone (n = 24), Central Dry Zone (n = 237), South-Eastern Dry Zone (n = 426), Southern Dry Zone (n = 114), Southern Transition Zone (n = 198), Northern Transition Zone (n = 159), and Hilly Zone (n = 48). Additionally, samples were grouped based on the year of collection: 2021 (n = 513), 2022 (n = 462), 2023 (n = 276), and 2024 (n = 102).

A chi-square analysis was conducted using GraphPad Prism 8.0.1 to assess the association between categorical variables and the proportion of positive cases for 451 samples each of blood, nasal swabs, and skin tissue or scab. The chi-square test for proportions was applied to evaluate statistical significance and determine whether a significant association exists between the analyzed variables and the percentage of positive cases.

Statistical analyses were conducted using the Statistical Package for the Social Sciences (IBM^®^ SPSS^®^ version 27). In this study, clinical samples of lumpy skin disease (LSD), including nasal swabs, whole blood, and skin nodules or scabs, acted as dependent variables, representing the diagnostic outcomes. Independent variables (predictors) included various risk factors such as agro-climatic region, age, sex, breed, and year of sample collection, as these factors could influence disease occurrence or be associated with diagnostic results.

A univariate analysis was initially performed to evaluate each risk factor individually. This was followed by a multivariate logistic regression analysis to assess the combined effects of multiple risk factors on disease outcomes. In the logistic regression model, the odds ratio, also known as Exp(B), was calculated to quantify the strength and direction of associations between predictor variables and disease outcomes. A 95% confidence interval (CI) was estimated to determine the likely range of the true population parameter, helping assess uncertainty and precision. Statistical significance was determined using a p-value threshold of < 0.05, indicating a significant association, while a p-value ≥ 0.05 suggested no statistically significant relationship.

## 3 Results

### 3.1 Epidemiology of LSD

**Figure 1:**
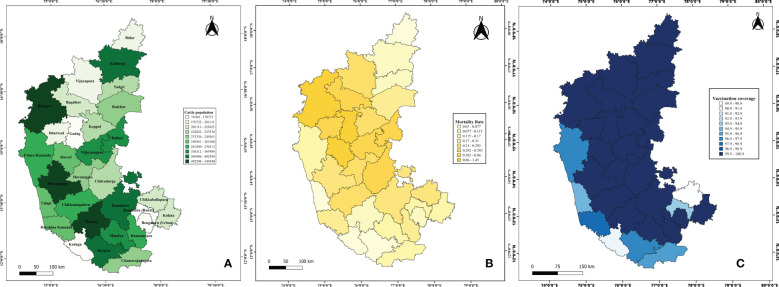
Demographic representation of epidemiological data on LSD in Karnataka. The figure illustrates the gradient distribution of **(A)** cattle population highlighting regions with high and low livestock concentrations; **(B)** Mortality rate; and **(C)** Vaccination coverage.

According to the 20th Livestock Census (2017), Karnataka's cattle and buffalo population is approximately 12 million, representing 4.3% of the national livestock population. The total cattle population in the state is 8,469,004, with the highest population observed in districts such as Belagavi (549,540), Hassan (548,185), Shivamogga (518,653), and Mysore (492,598). Conversely, districts with the lowest cattle populations include Kodagu (71,684), Gadag (136,311), Dharwad (172,219), and Bidar (173,634) (**Figure 1A**).

**Figure 2:**
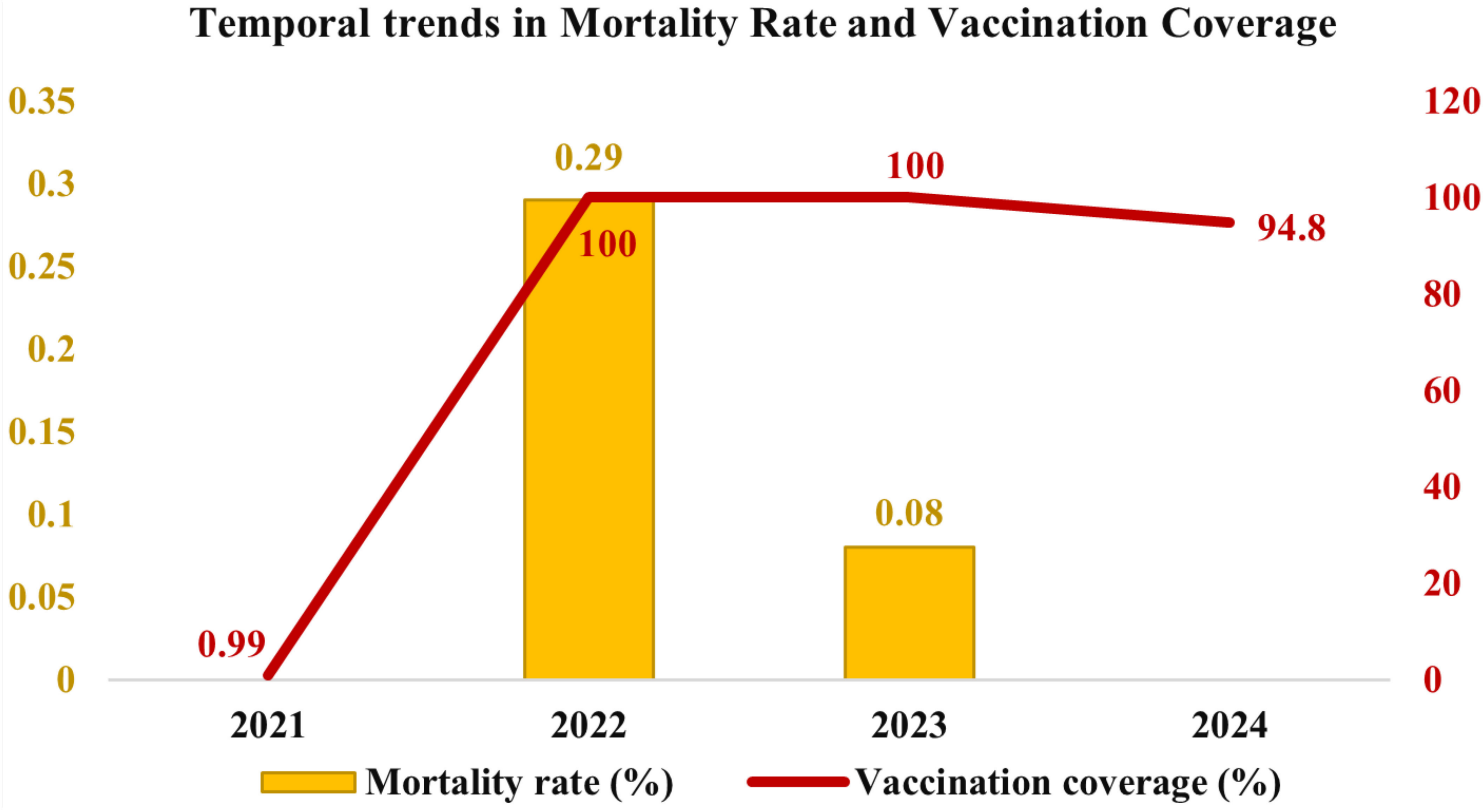
Temporal distribution of mortality rate and vaccination coverage in cattle in Karnataka from 2021 to 2024. The graph represents the mortality rate (in percentage) with yellow bars (left Y-axis), while the red line indicates vaccination coverage (in percentage) (right Y-axis).

Vaccination coverage data from 2021 to 2024 indicated that several districts, including Bengaluru Urban, Chitradurga, Davangere, Kolar, Ramanagara, Shivamogga, Tumakuru, Chikkamagaluru, Mandya, and others, achieved 100% vaccination coverage in 2022. However, vaccination coverage varied across the years, with an initial rate of 0.99% in 2021, peaking at 100% in 2022 and 2023, followed by a slight decline to 94.8% in 2024 (**Figure 2**). Additionally, districts such as Bengaluru Rural (93.89%), Chikkaballapur (89.87%), Chamarajanagar (96.42%), Dakshina Kannada (97.95%), Mysuru (96.92%), Udupi (95.71%), Uttara Kannada (97.51%), and Kodagu (91.24%) exhibited slightly lower vaccination coverage (**Figure 1C**).

The disease spread across 4,380 villages in 160 taluks, affecting over two million cattle, spanning 30 districts of Karnataka. Mortality rates from 2021 to 2024 indicated that districts such as Belagavi (1.45%), Gadag (1.07%), and Haveri (1.15%) exhibited higher mortality rates. In contrast, Kodagu (0.03%), Udupi (0.04%), Mysuru (0.07%), and Bidar (0.07%) reported the lowest mortality rates (**Figure 1B**). A temporal analysis of mortality rates revealed no reported fatalities in 2021, followed by an increase to 0.29% in 2022, a subsequent decline to 0.08% in 2023, and no reported mortality rate in 2024 (**Figure 2**).

**Figure 3:**
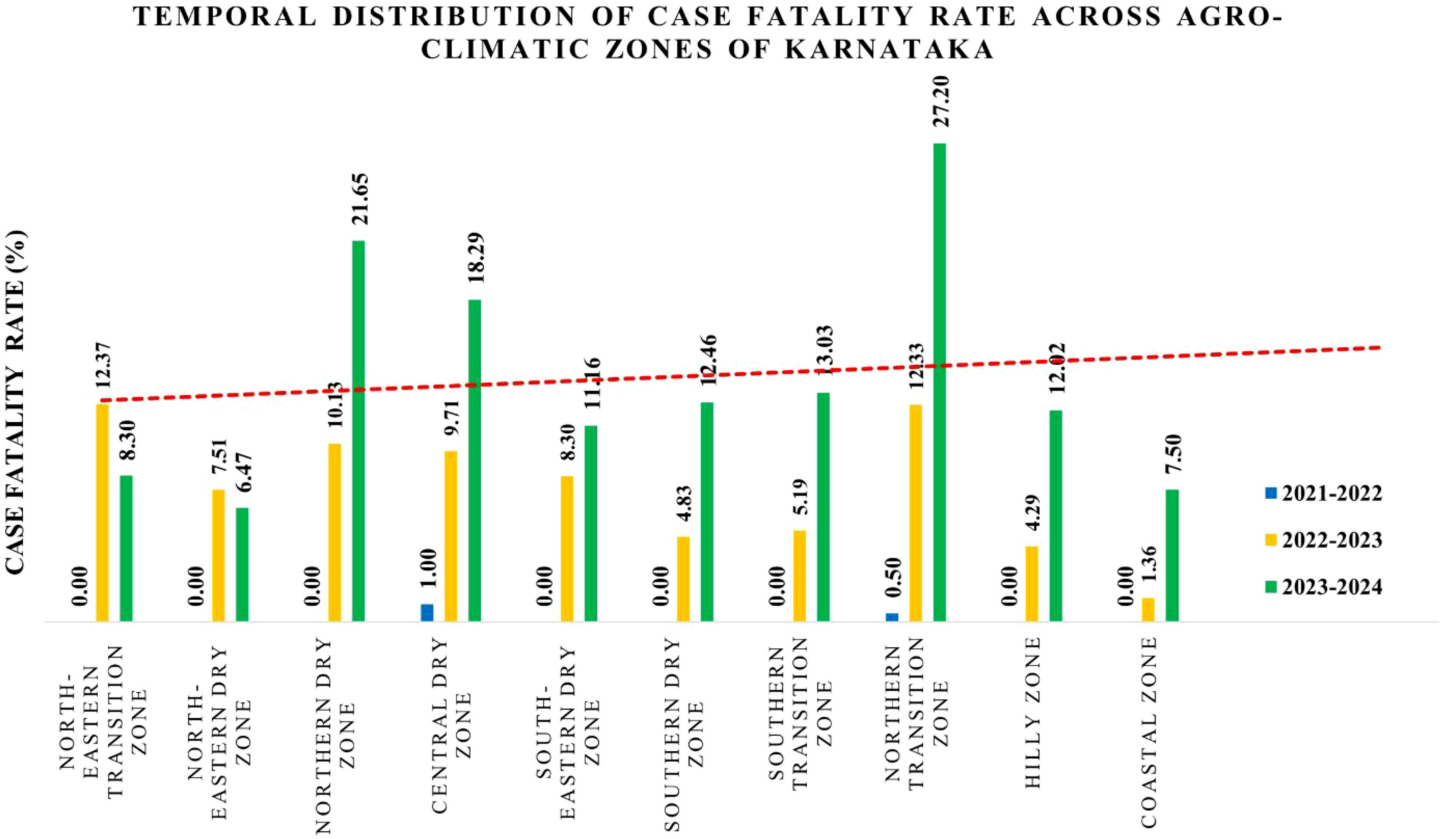
Graphical representation of case fatality rate across agro-climatic zones of Karnataka. The bar graph represents the increasing trend in CFR over three time periods: 2021–2022, 2022–2023, and 2023–2024. The X-axis denotes the agro-climatic zones, while the Y-axis represents the case fatality rate.

The case fatality rate (CFR) across Karnataka's agro-climatic zones from 2021 to 2024 exhibited distinct temporal variations (**Figure 3**). During 2021–2022, the overall CFR was 0.15%, with most zones reporting no fatalities, except for the Central Dry Zone (1%) and the Northern Transition Zone (0.5%). By 2022-2023, the CFR increased substantially to an average of 7.6%, with the highest rates recorded in the North Eastern Transition Zone (12.37%), Northern Transition Zone (12.33%), Northern Dry Zone (10.13%), and Central Dry Zone (9.71%). The Southern Dry Zone (4.83%) and Coastal Zone (1.36%) exhibited the lowest CFR during this period (**Figure 3**).

In 2023–2024, the CFR further increased to an average of 13.8%, with certain regions experiencing pronounced spikes. The Northern Transition Zone, including Belagavi and Haveri districts, recorded the highest CFR at 27.20%, aligning with their elevated mortality rates in 2022. Similarly, the Northern Dry Zone (21.6%) and Central Dry Zone (18.29%) showed substantial increases. The North Eastern Transition Zone, which had the highest CFR (12.37%) in 2022-2023, exhibited a 4.07% decline in 2023-2024. Meanwhile, the North Eastern Dry Zone maintained a relatively lower CFR of 6.47%, whereas the Coastal Zone (7.50%), Hilly Zone (12.02%), and Southern Transition Zone (13.03%) continued to show an upward trend (**Figure 3**).

### 3.2 Spatial statistics

#### 3.2.1 Moran's І scatter plot analysis

**Figure 4:**
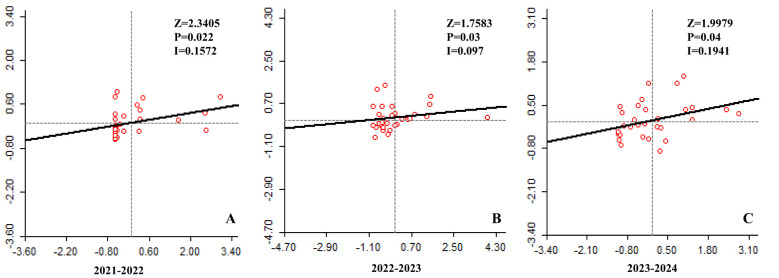
Moran's І scatter plots of LSD cases in Karnataka. The scatter plot illustrates the spatial correlation between the number of LSD cases in each district (represented on x-axis) and the number of cases in adjacent districts (represented on y-axis) for the years **(A)** 2021-2022, **(B)** 2022-2023, and **(C)** 2023-2024.

The spatial correlation of LSD cases in Karnataka using Moran's I indices for the time periods 2021-2022, 2022-2023, and 2023–2024 were shown in **Figures 4A–C** respectively. The analysis assessed the association between the number of LSD cases in each district and those in neighboring districts. For all three time periods, Moran's I indices were greater than zero, indicating significant spatial clustering of LSD cases. The calculated Moran's I values were 0.1572 (Z-score = 2.3405, p = 0.022) for 2021-2022, 0.097 (Z-score = 1.7583, p = 0.03) for 2022-2023, and 0.1941 (Z-score = 1.9979, p = 0.04) for 2023-2024. A statistically significant positive spatial association showed that districts with a higher number of LSD cases tend to be surrounded by neighboring districts with similarly high numbers of cases.

#### 3.2.2 Local indicators of spatial association clustering

**Figure 5:**
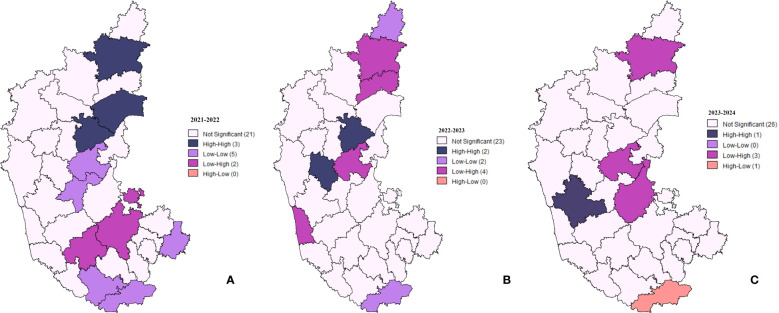
Spatial clustering and outliers of LSD cases in Karnataka. The maps depict LISA clustering for three time periods: **(A)** 2021-2022, **(B)** 2022-2023, and **(C)** 2023-2024, highlighting spatial variations and trends over time.

Local Indicators of Spatial Association (LISA) clustering analysis was performed to examine the spatial patterns and clustering of lumpy skin disease (LSD) cases across districts in Karnataka (**Figure 5**). Spatial clustering of different districts in Karnataka for the years 2021-2022, 2022-2023, and 2023-2024, where high-high clusters, depicted in dark blue, represent districts with a high number of cases surrounded by neighboring districts with similarly high case numbers. Low-low clusters, shown in purple, indicate low numbers of cases in a district containing low numbers of cases in neighboring districts. High-low outliers, represented in peach, correspond to districts with a high number of cases surrounded by neighboring districts with low numbers of cases. Conversely, low-high outliers, shown in lilac, represent districts with a low number of cases containing high numbers of cases in neighboring districts.

During the 2021–2022 period (**Figure 5A**), three districts exhibited High-High clustering, indicating that they had significantly elevated case counts and were surrounded by similarly high-value districts. Five districts displayed low-low clustering, representing areas with consistently lower-case numbers. Additionally, two districts were classified as low-high, signifying regions with low case values neighboring high-case districts, while one district exhibited high-low clustering, indicating an isolated high-case district surrounded by low-case regions.

In 2022–2023, the number of High-High clustering districts decreased to two, suggesting a reduction in spatial clustering of regions with a high number of cases (**Figure 5B**). The low-low clustering pattern diminished from five districts to zero, implying a more dispersed distribution of low-case areas. Conversely, the number of low-high clustering districts slightly increased to four.

By 2023–2024, a notable shift was observed, with only one district remaining in the High-High clustering category (**Figure 5C**). Low-Low clustering completely disappeared, indicating the absence of well-defined clusters of low-case districts. Additionally, high-low clustering was identified in one district, representing an isolated high-case region among districts with lower case counts.

#### 3.2.3 Gi* (Getis-Ord-Gi*) statistic

**Figure 6:**
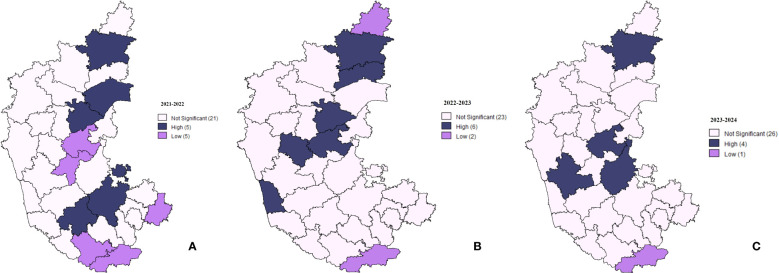
Gi* statistics for the LSD in Karnataka. The cold and hot spots clusters for different districts in Karnataka for the years **(A)** 2021-2022, **(B)** 2022-2023, and **(C)** 2023-2024.

Getis-Ord Gi* spatial analysis was computed to assess the distribution of high and low spatial clusters of lumpy skin disease (LSD) cases across districts in Karnataka for the years 2021–2022, 2022–2023, and 2023–2024 (**Figure 6**), where dark blue districts exhibit significantly high Gi* values, indicating hotspots where LSD cases were concentrated. Purple shows districts with significantly low Gi* values, representing cold spots with fewer cases. Light pink shows districts that do not show statistically significant clustering patterns. Using a 95% confidence level, statistically significant hotspots (high-case clusters) and cold spots (low-case clusters) of LSD incidence were identified. In 2021–2022, five districts exhibited high-risk disease clustering, indicating localized hotspots of LSD cases, while five districts demonstrated low-risk clustering, representing regions with significantly lower-case incidence (**Figure 6A**). By 2022–2023, the number of high-risk clusters increased to six, suggesting an expansion in disease clustering, whereas low-risk clusters decreased to two, indicating a decline in areas with significantly lower-case concentrations as shown in **Figure 6B**. In 2023–2024, the number of high-risk clusters reduced to four, suggesting a weakening of spatial clustering patterns, with most districts exhibiting non-significant spatial trends. Additionally, a single persistent low-risk cluster was observed, indicating further dispersion of the disease across the region (**Figure 6C**).

### 3.3 Molecular detection and its descriptive statistics

**Figure 7:**
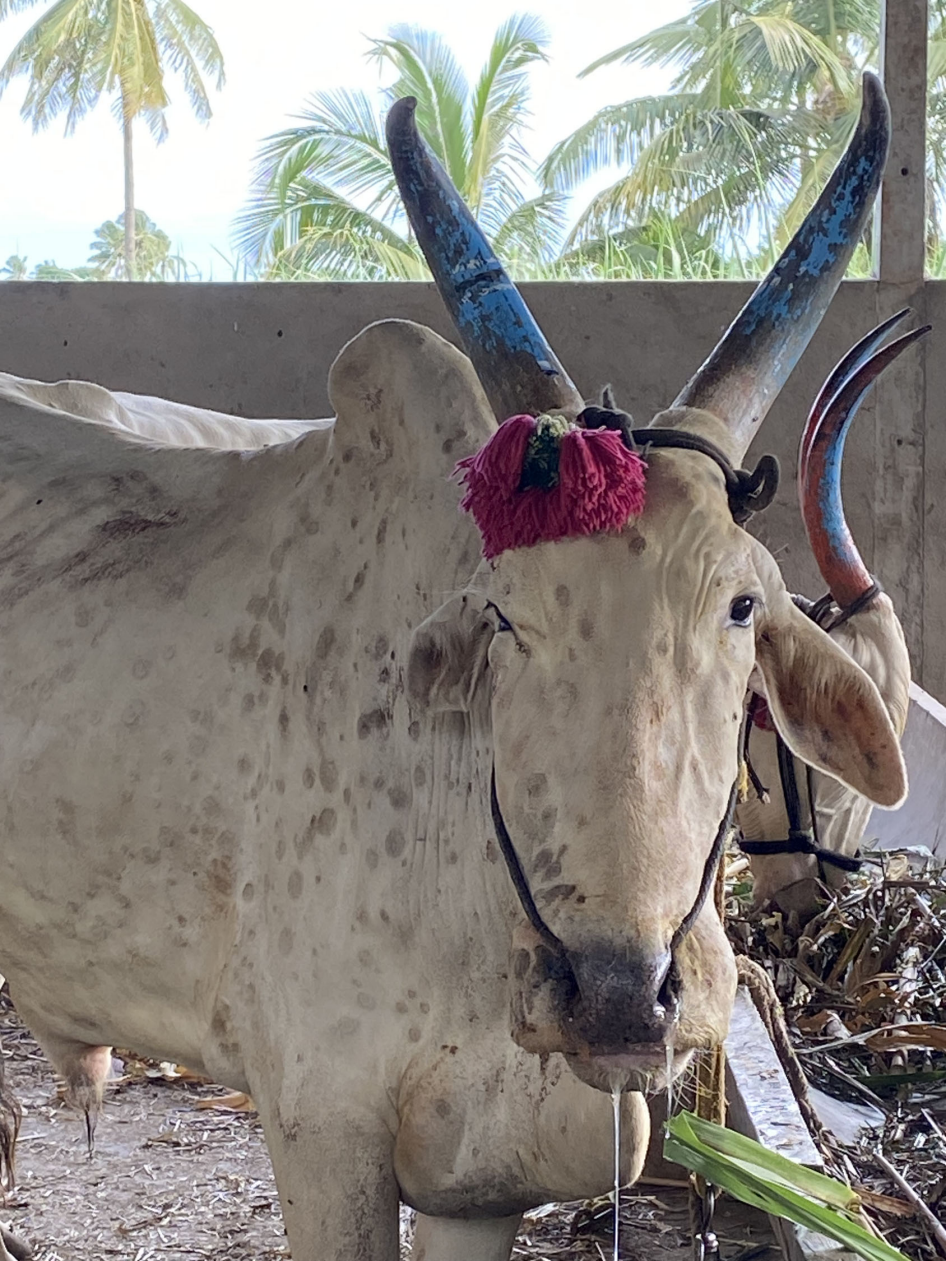
Clinical manifestation of LSD in cattle. Cattle affected by LSD exhibit multiple skin nodules, a characteristic sign of LSDV infection.

At the peak of the LSD outbreaks, an extensive epidemiological investigation was conducted in the affected areas of Karnataka. Infected cattle showed typical clinical signs of LSD, such as an elevated body temperature, reduced milk production, anorexia, lymphadenopathy, lacrimation, hypersalivation, and characteristic nodular skin lesions about 50 mm in diameter (**Figure 7**). Diagnostic evaluation using PCR demonstrated variations in positivity across different sample types. Statistical analysis using the Chi-square test demonstrated a significant association between sample type and PCR outcome with a Pearson chi-square value of χ² = 6.438 (df = 2, p = 0.040) and a likelihood ratio test result of χ² = 6.409 (df = 2, p = 0.041). Among the tested samples, skin nodules or scabs showed the highest positivity rate at 83.6%, followed by nasal swabs (80.9%) and blood samples (76.9%) within the category.

### 3.4 Virus isolation

**Figure 8:**
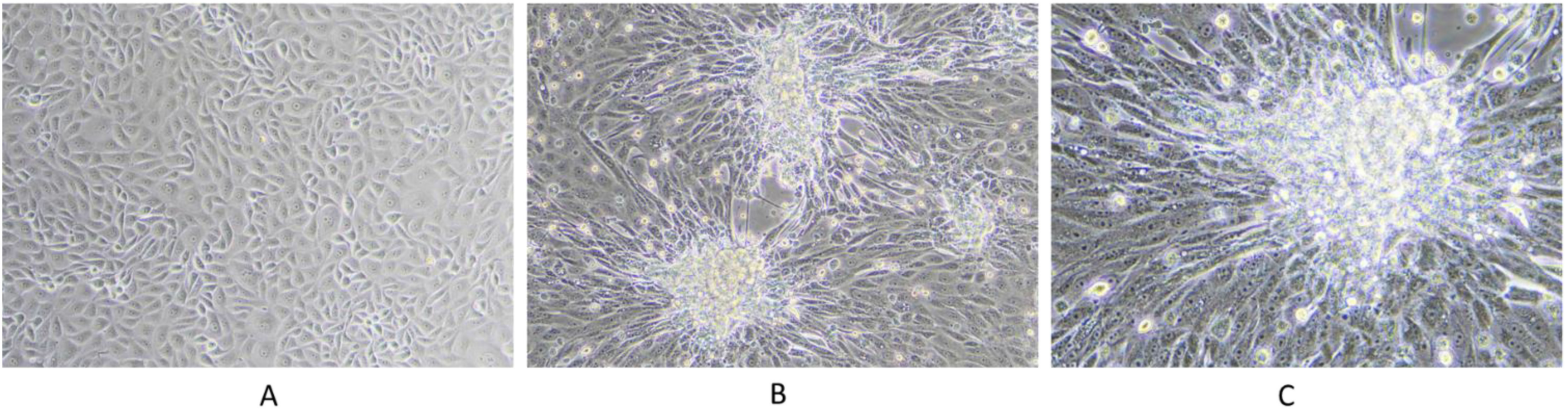
LSDV infection in MDBK cell line. The figure illustrates the cytopathic effects of LSDV in the MDBK cell line: **(A)** control cells; **(B)** infected cells at 5 dpi at 10x magnification, showing foci formation; and **(C)** infected cells at 5 dpi at 20x magnification, highlighting viral syncytium formation.

Representative samples, including scabs and nasal swabs collected during LSD outbreaks, were used for virus isolation. The isolates were subjected to two to three blind passages to induce characteristic cytopathic effects (CPE) in cell cultures. The observed CPE, indicative of LSDV infection, included the formation of multinucleated syncytia, cellular rounding, vacuolation, and ballooning, culminating in cytolysis and cell detachment. The presence of foci, characterized by clusters of infected cells, indicated robust viral replication. Consistently, the LSDV field isolates were successfully adapted to the MDBK cell line, with CPE becoming apparent between the sixth and tenth passages. This progressive adaptation and foci formation, as illustrated in **Figure 8** underscores the pronounced cytopathic nature of LSDV, reflected in distinct morphological alterations in infected cells (**Figure 8**).

### 3.5 Phylogenetic analysis

**Figure 9:**
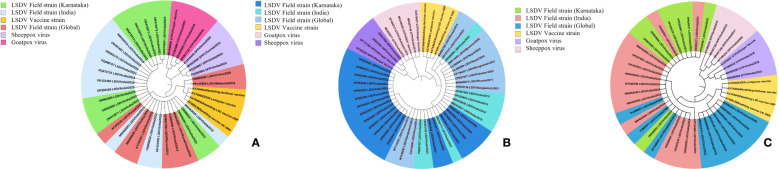
Phylogenetic analysis of full-length genes. Phylogenetic tree of **(A)** *P32*, **(B)** *RPO30*, and **(C)** *GPCR* genes of LSDV isolates from cattle in Karnataka, India.

For the molecular epidemiology of the disease, cell culture isolated LSDV from affected cattle, and along with PCR-positive diagnostic samples, full-length sequencing and phylogenetic analysis were employed on key genes, including *P32* (envelope protein), *RPO30* (RNA polymerase gene), and *GPCR* (integral membrane protein). The sequences were edited and submitted to GenBank and received accession numbers for *P32*, *GPCR*, and *RPO30*. Maximum likelihood phylogenetic trees were constructed, yielding log likelihood values of -1489.21 for *P32*, -942.71 for *RPO30*, and -2096.13 for *GPCR*, as illustrated in **Figures 9A–C** respectively. The analysis revealed distinct clustering patterns, depicting the genetic relationships and evolutionary trajectory of LSDV over time. Karnataka isolates are distributed across multiple clades, indicating regional dissemination and genetic variability. These isolates exhibited close phylogenetic clustering with LSDV field strains circulating in India, Kenya, Ismailia, Serbia, Russia, Israel, Turkey, Kazakhstan, Bangladesh, Pakistan, Nepal, Myanmar, and neighboring regions of Karnataka, suggesting extensive geographical transmission networks. Notably, Karnataka isolates demonstrated phylogenetic proximity to Middle Eastern and African strains, particularly from Kenya and Egypt, suggesting historical transmission links. Meanwhile, recent isolates exhibited slight genetic divergence, indicative of local adaptation and ongoing viral evolution. The phylogenetic trees also clearly differentiated field isolates from vaccine strains, with vaccine-derived LSDV forming distinct clades in each tree, emphasizing the genetic divergence between wild-type and vaccine strains. Furthermore, Karnataka LSDV isolates were distinctly separated from other Capripoxviruses, such as goat pox and sheep pox viruses, reaffirming LSDV's genetic specificity within the Capripoxvirus genus and its unique evolutionary trajectory as a distinct lineage (**Figure 9**).

### 3.6 LSD-associated risk-factors

The univariate and multivariate regression analyses revealed several demographic risk factors associated with the prevalence of LSD in Karnataka, with both sets of analyses revealing key findings summarized in **Tables 2** and **3**. Age emerged as a significant determinant in both analyses. Cattle aged 1 to 5 years and those less than one year showed a higher risk of LSD infection compared to cattle aged over 5 years. Specifically, cattle in the age group of 1 to 5 years exhibited 2.5–3.5 times greater odds of infection, a trend that persisted in multivariate models. Further, younger cattle (<1 year) demonstrated a significantly moderate risk of infection (OR: 0.951-2.017) when compared to cattle aged over 5 years.

**Table 2:** Shows univariate logistic regression analyses for different risk factors such as zones, sex, age, breed, and year with different sample types, namely blood, nasal swabs, and biopsy with different statistical parameters in Karnataka.

Risk factors		Whole blood		Nasal swab		Skin nodule/scab	
		OR	95% CI	OR	95% CI	OR	95% CI
Zones	*p*-value	0.001^*^		0^*^		0^*^	
	North Eastern Dry Zone	2.5	0.284 - 22.042	1.705	0.184 - 15.782	0	0
	Northern Dry Zone	2.143	0.116 - 39.469	2.143	0.116 - 39.469	2.143	0.116 - 39.469
	Central Dry Zone	2.955	0.358 - 24.365	1.233	0.138 - 11.002	1.233	0.138 - 11.002
	South-Eastern Dry Zone	4.541	0.578 - 35.679	4.189	0.532 - 32.968	3.85	0.488 - 30.353
	Southern Dry Zone	3.387	0.381 - 30.086	1.286	0.124 - 13.382	0.833	0.070 - 9.900
	Southern Transition Zone	12.5	1.559 - 100.198	10.385	1.294 - 83.362	10.385	1.294 - 83.362
	Northern Transition Zone	4.39	0.525 - 36.722	4.39	0.525 - 36.722	2.667	0.308 - 23.110
	Hilly Zone	1	−	1	−	1	−
Sex	*p*-value	0.029^*^		0.065		0.042^*^	
	Female	1.69	1.055 - 2.708	1.61	0.971 - 2.668	1.762	1.021 - 3.040
	Male	1	−	1	−	1	−
Age	*p*-value	0^*^		0.001^*^		0.001^*^	
	<1 year	0.951	0.5 - 1.809	1.087	0.547 - 2.162	2.017	0.971 - 4.191
	1 year - 5 year	2.597	1.557 - 4.331	2.576	1.481 - 4.481	3.455	1.829 - 6.528
	≥5 year	1	−	1	−	1	−
Breed	*p*-value	0.003^*^		0.062		0.01^*^	
	Indigenous breed	0.986	0.477 - 2.039	0.938	0.440 - 1.998	0.920	0.519 - 3.084
	Exotic breed	2.139	1.069 - 4.283	1.684	0.816 - 3.478	2.591	1.105 - 6.075
	ND	1	−	1	−	1	−
Year	*p*-value	0.008^*^		0.001^*^		0^*^	
	2021	3.314	1.138 - 10.714	3.229	1.025 - 9.798	12.055	0.018 - 108.172
	2022	2.365	1.302 - 5.464	2.425	1.189 - 6.957	3.481	0.215 - 6.074
	2023	1.722	1.313 - 3.668	1.022	0.513 - 2.668	1.684	0.295 - 3.585
	2024	1	−	1	−	1	−

Sig., significant (* p < 0.05 = significant); OR, odds ratio; and CI, confidence interval.

**Table 3:** Shows multivariate logistic regression analyses for different risk factors such as agro-climatic zones, sex, age, breed, and year of collection with different sample types, namely blood, nasal swabs, and biopsy with different statistical parameters in Karnataka.

Risk factors		Whole blood			Nasal swab		Skin nodule/scab	
		OR		95% CI	OR	95% CI	OR	95% CI
*p*-value		0.034*			0.002*		0.003*	
Zones	North Eastern Dry Zone	5.397	0.554 - 52.606		2.921	0.28 - 30.457	0	
	Northern Dry Zone	4.867	0.234 - 101.123		5.098	0.243 - 106.795	4.021	0.662 - 89.574
	Central Dry Zone	4.655	0.505 - 42.881		1.553	0.152 - 15.865	2.72	0.262 - 28.262
	South-Eastern Dry Zone	4.855	0.595 - 39.639		5.090	0.618 - 41.929	4.631	0.561 - 38.624
	Southern Dry Zone	5.802	0.598 - 56.287		2.047	0.179 - 23.427	2.202	0.165 - 29.392
	Southern Transition Zone	14.481	1.699 - 123.44		12.848	1.488 - 110.96	17.998	0.662 - 489.574
	Northern Transition Zone	5.663	0.638 - 50.302		5.882	0. 655 - 52.814	4.654	0.497 - 43.584
	Hilly Zone	1	−		1	−	1	−
*p*-value		0.764			0.852		0.492	
Sex	Female	1.42	0.457 - 1.778		1.070	0.525 - 2.180	1.322	0.596 - 2.934
	Male	1	−		1	−	1	−
*p*-value		0.001*			0.016*		0.039*	
Age	<1 year	1.085	0.347 - 1.777		1.252	0.287 - 1.740	1.252	0.465 - 3.375
	1 year - 5 year	2.301	1.321 - 4.006		1.914	1.042 - 3.515	2.409	1.154 - 5.029
	≥5 year	1	−		1	−	1	−
*p*-value		0.169			0.314		0.377	
Breed	Indigenous breed	0.948	0.377 - 2.388		0.480	0.172 - 1.344	0.571	0.159 - 2.054
	Exotic breed	1.722	0.752 - 3.943		1.745	0.289 - 1.918	1.023	0.324 - 3.232
	ND	1	−		1	−	1	−
*p*-value		0.11			0.009*		0	
Year	2021	5.053	1.772 - 14.411		5.37	1.825 - 15.798	21.338	5.559 - 81.903
	2022	2.661	1.008 - 7.022		3.525	1.302 - 9.539	3.014	1.111 - 8.179
	2023	2.085	0.754 - 5.764		2.085	0.751 - 5.791	1.707	0.605 - 4.817
	2024	1	−		1	−	1	−

Sig., significant (* p < 0.05 = significant); OR, odds ratio; and CI, confidence interval.

Breed analysis indicated that exotic cattle were more susceptible to LSDV infection compared to indigenous cattle. In univariate analysis, exotic breeds had 2.1–2.6 times higher odds of infection compared to indigenous breeds, a finding corroborated by multivariate regression. Gender differences were evident, with females showing a higher likelihood of infection than males. This trend was particularly notable in all the different types of samples, where females showed 7% to 76% more chance of LSD incidence than males. Geographical location significantly influenced LSD prevalence. The Southern Transition Zone covering Chikkamagaluru, Hassan, and Shivamogga districts exhibited the highest infection risk across all sample types. Blood samples from this zone had 12.5 times (OR: 12.5; 95% CI: 1.559 - 100.198) greater odds of testing positive compared to the Hilly Zone, with nasal swabs and skin samples also showing elevated risks. Conversely, the Northern Dry Zone was identified as a low-risk area, with significantly reduced odds in both univariate and multivariate analyses. Temporal analysis revealed a marked reduction in LSD risk over time, with 2021 representing the peak outbreak year. Skin scab samples collected in 2021 showed 21.3 times greater odds of infection (OR: 21.338; 95% CI: 5.559–81.903) compared to 2024. Subsequent years, including 2022 and 2023, showed a progressive decline in infection risk, reflected by steadily decreasing odds.

## 4 Discussion

Lumpy Skin Disease (LSD) is an emerging transboundary pox viral disease that affects bovines, including cattle and buffalo. Belonging to the *Chordopoxvirinae* subfamily within the *Poxviridae* family, LSD has gained global significance due to its rapid spread and severe economic repercussions. Recognizing its impact, the World Organization for Animal Health (WOAH) has classified LSD as a "notifiable transboundary disease." The outbreaks of LSD have substantial economic consequences, affecting both micro- and macroeconomic sectors. The disease significantly reduces milk production and, in severe cases, leads to mortality, thereby disrupting the dairy industry and national economic stability. Furthermore, irreversible damage to skin and hides results in financial losses in the leather industry, while the death of working animals, such as bulls and oxen, negatively affects agricultural productivity by delaying land preparation and decreasing crop yields. Additionally, the disease disrupts herd dynamics, reducing the availability of surplus livestock.

Our epidemiological analysis of LSD outbreaks in Karnataka highlighted the critical role of vaccination and control measures in reducing disease prevalence. The disease metrics study showed high-density livestock regions such as Belagavi, Shivamogga, and Tumkur required extensive vaccination efforts. The analysis of disease metrics indicated a decline in LSD-related mortality rates over time, demonstrating the effectiveness of disease management strategies. A strong correlation was observed between livestock density, mortality rates, and vaccination coverage, with higher vaccination rates associated with lower mortality. However, population density alone did not determine mortality trends, as evidenced by variations between districts with similar cattle populations, such as Hassan (548,185 cattle, 0.11% mortality rate) and Shivamogga (518,653 cattle, 0.27% mortality rate). The overall decline in mortality rates from 2021 to 2024 suggests that vaccination, biosecurity measures, and natural herd immunity played a pivotal role in disease control. However, the increasing case fatality rate (CFR) indicates that a greater proportion of infected animals succumbed to the disease, potentially due to increased virulence, delayed treatment, or host susceptibility factors. The highest CFR (27.2%) was recorded in the Northern Transition Zone in 2022–2023, indicating regional variations in disease severity. These findings align with previous studies on LSD incidence in Karnataka (Prabhu et al., 2023) and suggest that additional demographic and environmental factors influence LSD dynamics. The necessity for region-specific control strategies is evident, as uniform approaches may not be equally effective across different regions.

The study also examined the spatial-temporal distribution of LSD outbreaks in Karnataka from 2021 to 2024 using advanced statistical techniques such as Moran's I, Local Indicators of Spatial Association (LISA) clustering, and Getis-Ord Gi* hotspot analysis. The Moran's I coefficients (ranging from 0.097 to 0.1941) indicated a positive spatial autocorrelation, suggesting clustering of LSD cases in districts with high incidence rates. Permanent hotspots identified in Kalaburagi, Bellary, Chitradurga, and Shivamogga highlight the need for sustained disease monitoring while consistently low-incidence areas like Chamarajanagar suggest effective containment measures.

Furthermore, LSD case distribution exhibited a shifting trend over time, with high-case clusters initially concentrated in the northeastern and northern dry zones in 2021–2022 before gradually extending towards the central and hilly zones by 2023–2024. Environmental factors such as humidity, rainfall, and vector migration might have played a significant role in disease spread. The role of blood-feeding arthropods in LSDV transmission was evident, with spatial clustering trends indicating higher case densities in vector-prone areas. These findings emphasize the necessity of integrating targeted vector control strategies with vaccination programs to enhance disease management.

Our study also evaluated the diagnostic efficacy of different sample types for LSDV detection. PCR-based detection demonstrated the highest sensitivity, particularly in nodule and nasal swab samples, which provided consistent viral presence across various infection stages. This underscores the importance of early detection and the role of non-invasive sampling methods in surveillance programs. Phylogenetic analysis of Karnataka LSDV isolates revealed a close genetic relationship with Middle Eastern and African strains, suggesting long-term geographical spread facilitated by livestock movement and trade (Bayyappa et al., 2025). The genetic divergence observed in recent isolates highlights the virus's ongoing adaptation, reinforcing the need for continuous genomic surveillance to monitor emerging variants and assess vaccine efficacy.

The study also identified key risk factors influencing LSDV susceptibility. Cattle aged 1 to 5 years were found to be more vulnerable due to their developing immune systems, higher metabolic demands, and increased exposure risks within herds. Female cattle exhibited a higher predisposition, likely due to physiological stressors such as pregnancy and lactation, which can suppress immune responses. This aligns with findings from a previous study (Manjunathareddy et al., 2024; Manjunatha Reddy et al., 2025). Additionally, exotic breeds showed increased susceptibility compared to indigenous breeds, potentially due to their lack of natural resistance, thinner skin that makes them more prone to vector bites, and the physiological stress of high milk production (Sanjeevakumar et al., 2023). Epidemiological data indicated that the Southern Transition Zone had a high CFR (13.03%), which was further supported by regression analysis. This region experiences moderate to high temperatures and fluctuating humidity, creating ideal conditions for vector proliferation, particularly stable flies, mosquitoes, and midges, which are primary LSDV vectors. A similar year-wise trend was observed in both regression and spatial-temporal analyses.

The findings of this study provide a crucial foundation for refining LSD control policies, optimizing vaccine deployment, and implementing adaptive strategies tailored to regional risk factors. While vaccination has significantly contributed to disease mitigation, the rising CFR underscores the need for a comprehensive approach that integrates early diagnosis, vector control, and region-specific biosecurity measures.

Despite the valuable epidemiological and spatial insights provided by this study, certain limitations must be acknowledged. The analysis was based on reported cases, which may not fully capture underreported or asymptomatic infections. Variability in diagnostic capacities across regions could have influenced data accuracy. Furthermore, while environmental and climatic factors were considered, additional determinants such as socioeconomic conditions, livestock movement patterns, and farm management practices could further refine disease risk models. Although vaccine effectiveness was inferred from epidemiological data, controlled studies are required to assess immunological responses across different cattle breeds and age groups.

Future research should integrate environmental risk modeling with epidemiological data to enhance predictive disease management strategies. Advanced machine learning models incorporating climatic, demographic, and genomic data could improve early warning systems for LSD outbreaks. Expanding genomic surveillance to track viral evolution and vaccine efficacy will be essential for adapting control measures against emerging variants. Moreover, longitudinal studies on heterologous vaccine-induced immunity and their comparative effectiveness with homologous vaccines will help optimize vaccination strategies.

Additionally, exploring alternative control measures such as antiviral treatments, vector management strategies, and breed-specific resistance assessments will further strengthen LSD mitigation efforts. Public-private partnerships can facilitate the development of cost-effective vaccines and improved biosecurity protocols, ensuring sustainable disease control at both national and global levels.

## Data Availability

The datasets presented in this study can be found in online repositories. The names of the repository/repositories and accession number(s) can be found in the article/**Supplementary Material**.
