# Supplementary material for: Molecular epidemiological and spatiotemporal analysis of lumpy skin disease outbreaks in cattle from Karnataka, India
Source: Front Cell Infect Microbiol. 2025 Jun 24;15:1596973. doi: 10.3389/fcimb.2025.1596973 (PMC12234529; doi:10.3389/fcimb.2025.1596973)
Supplement: Supplementary file 1 [file Table1.docx]

Supplementary Material

# Supplementary Table

**Supplementary Table 1** shows the distribution of districts across the agro-climatic zones of Karnataka.

| **Agro-climatic Zones** | **Districts** |
| --- | --- |
| North Eastern Transition zone | Bidar |
| North Eastern Dry Zone | Bellary, Kalaburagi, Koppal, Raichur, Yadgiri |
| Northern Dry Zone | Bagalkote, Gadag, Vijayapura, Vijaynagara |
| Central Dry Zone | Chitradurga, Davanagere, Tumakuru |
| South-Eastern Dry Zone | Bengaluru, Bengaluru rural, Chikkaballapura, Kolar, Ramanagara |
| Southern Dry Zone | Chamarajanagara, Mandya, Mysuru |
| Southern Transition Zone | Chikkamagaluru, Hassan, Kodagu, Shivamogga |
| Northern Transition Zone | Belagavi, Dharwad, Haveri |
| Hilly Zone | Uttara Kannada |
| Coastal Zone | Dakshina Kannada, Udupi |
